# Mesoscale Model to Select the Ideal Location for New Vineyard Plantations in the Rioja Qualified Denomination of Origin

**DOI:** 10.1155/2014/403683

**Published:** 2014-10-16

**Authors:** E. Martínez-Cámara, J. Blanco, E. Jiménez, J. C. Saenz-Díez, J. Rioja

**Affiliations:** ^1^Department of Mechanical Engineering, University of La Rioja, 26004 Logrono, Spain; ^2^Department of Electrical Engineering, University of La Rioja, 26004 Logrono, Spain

## Abstract

La Rioja is the region where the top rated wines from Spain come from and also the origin of one of the most prestigious wines in the world. It is worldwide recognized, not only for the quality of the vine, but also for the many factors involved in the process that are controllable by the farmer, such as fertilizers, irrigation, etc. Likewise, there are other key factors, which cannot be controlled that play, however, a crucial role in the quality of the wine, such as temperature, radiation, humidity, and rainfall. This research is focused on two of these factors: temperature and irradiation. The objective of this paper is to be able to recognize these factors, so as to ensure a proper decision criterion when selecting the best location for new vineyard plantations. To achieve this objective, a mesoscale model MM5 is used, and its performance is assessed and compared using different parameters, from the grid resolution to the physical parameterization of the model. Finally, the study evaluates the impact of the different parameterizations and options for the simulation of meteorological variables particularly relevant when choosing new vineyard sites (rainfall frequency, temperature, and sun exposure).

## 1. Introduction

### 1.1. Research Context

La Rioja is the region that gives name to the most prestigious wines of Spain because of the wide diversity of soils and climates it has, which have allowed a great variety of wines with distinctive characteristics. Rioja wines are protected by Spain's oldest Denomination of Origin, officially recognized in 1925.

The Rioja Qualified Denomination of Origin (QDO) is located northwest of the depression of the Ebro River, bordering north with the Sierra Cantabria and south with the Sierra de la Demanda, spreading through the autonomous community of La Rioja and several municipalities of the Basque Country and Navarre, with a total of 63.593 hectares of vineyards currently protected by the QDO with which one of the most prestigious wines in the world is produced [1].

The quality of the products in the Rioja QDO is extremely high. The quality was chosen as the factor that characterizes Rioja wines under the QDO and has been the key to its success. The vine is an important factor, but it alone cannot guarantee a good grape production, let alone a good quality of wine. It is also necessary to consider the interaction of factors that influence the proper ripening of the fruit, which determine both the amount and quality of the vintage [2–10]. These factors are comprised in the following groups:

- permanent factors: soil, grape variety, density, and vineyard conformation;
- variable factors: temperature, irradiation, humidity, and rainfall frequency;
- accidental factors: pests, diseases, and weather issues;
- modifiable factors: pruning, fertilizers, irrigation, cultivation labors, pesticide treatments, and so forth.

In this paper, the variable factors are researched. The aim of the research is to be able to predict weather factors in any area of the Rioja QDO, in order to have a decision criterion when making new vineyard plantations and hence achieve the best product quality with an extensive knowledge of the variable weather factors.

The proposed model analyzes the following permanent factors [11].

*(i) Sun Exposure*. The geographic situation of the vineyard determines both temperature and light exposure in the zone. The minimum hours of annual sun exposure required for the cultivation of the vine range from 1500 to 1600 hours, out of which, at least 1200 must be received in the vegetative period.

*(ii) Temperature*. The temperature is a key factor for the proper vegetative development and obtainment of a complete ripening of the grapes. In the winter rest period, the dormant buds do not freeze until −12°C and the limbs as well as the stem resist up to −16°C. The temperature significantly affects the maturation of the grapes. It affects also the respiration level, making an impact on the substrates, sugars, organic acid levels, and so forth. Thus, the grape composition varies as the average maturation temperature does. This temperature must reach at least 18°C to achieve a satisfactory maturation level, being necessary, at least, a minimum of 15 days with air temperature above 30°C to obtain a good vintage.

*(iii) Rainfall Frequency*. The abundance or scarcity of water is important to the viability of the vine, for the proper development of the fruit and wine quality. In this concrete case, it is not particularly relevant to the study, as there is the possibility of irrigation in the dry season; therefore, it will not be treated deeply.

### 1.2. Mesoscale and Weather Forecasting Applied to Agriculture

The fifth generation mesoscale model of PSU/NCAR (MM5) [12] is a numerical model of a limited area, nonhydrostatic with sigma coordinates, and with field monitoring designed to simulate or predict atmospheric circulations on the mesoscale level, and it has been applied in several fields such as weather forecasting [13–18] or air quality studies [12, 19–25]. Nowadays, the mesoscale models have been evaluated in special ranges from tens to hundreds of miles on atmospheric variables such as wind, rain, and so forth [26], as well as in specific weather events such as hurricanes, heavy rains, and floods [27–31].

In the field of agriculture, there are studies that use models to predict weather. Rimmer et al. [32] showed that the rainfall needs to balance the irrigation for corn plantations, apples, and alfalfa. Katata et al. [33] studied the possible variations of moisture based on the existing mist in a particular area. Moreover, Dodla and Ratna [34] used MM5 for weather advisory on plantations in India. The study of Silva et al. [35], about the replacement of fixed weather stations by meteorological models in the basin of the Maipo River (Chile), is also noteworthy. Prabha and Hoogenboom [36] created a local mesoscale weather information model that could be used as a guideline for crop protection to effectively manage and mitigate the effects of frost damage. Jones and Thornton [37] describe a generalised downscaling and data generation method that takes the outputs of a general circulation model and allows the stochastic generation of daily weather data that are to some extent characteristic of future climatologies. Lee et al. [38] showed the application of CFD in the external atmospheric processes as well as modeling in land and water management. Finally, it is worth noting the contribution of Skelsey et al. [39] who used mesoscale models to predict the distribution of potato late blight spores caused by changes in wind direction and its intensity. Gärtner et al. [40] developed a simple downscaling approach based on available georeferenced farm census data and Swiss land use statistics.

Throughout the research, no specific relevant documentation has been detected in the field of modeling sites for new vineyard plantations, but, as mentioned in the previous paragraph, it is possible to use this methodology as a source of information on any of the variable factors that influence the maturation of the vine, which will thus determine the size and quality of the vintage. In this paper, the analysis will be focused on its application to the viticulture area in specific locations where the new plantations are established, taking always into account the relevant atmospheric variables for the cultivation of the vine.

Finally, the study evaluates the impact of the different parameterizations and options for the simulation of meteorological variables particularly relevant when choosing new vineyard sites (rainfall frequency, temperature, and sun exposure). The applications of the model are validated using different parameterizations and options at a local scale in a particular area of Spain (La Rioja).

## 2. Materials and Methods

### 2.1. Initialization of the Model and the Domains

**Figure 1:**
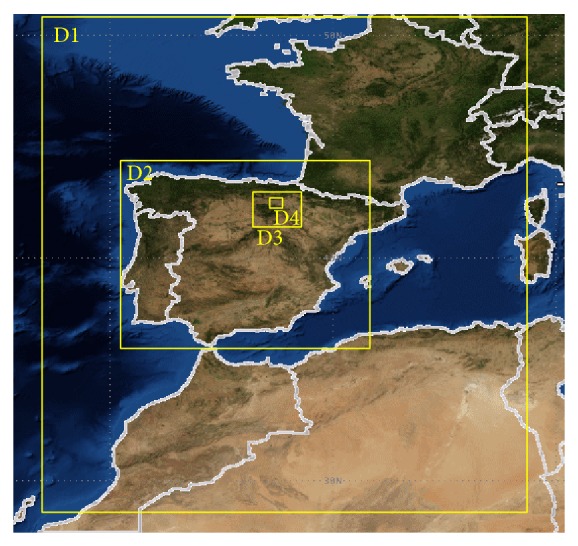
MM5 base-case model domains.

The base-case model domains are shown in Figure 1. The MM5 model has been built on a mother domain (D1) with 27 km of spatial resolution and centered on 40°N 4°W. This domain covers the Iberian Peninsula, as well as part in Europe and Africa, with the objective of capturing the synoptic characteristics and circulation patterns of the region. The first nested domain (D2), with a spatial resolution of 9 km, covers the Iberian Peninsula almost completely. The second nested domain (D3), with a spatial resolution of 3 km, covers the autonomous community of La Rioja. Finally, the innermost domain (D4) is centered on the meteorological towers used in the study and is composed of 34 columns and 49 rows of cells of a 1 × 1 km. The three nested domains interact with each other through a strategy of nesting in a single direction. The vertical structure of the model includes 23 layers that cover the entire troposphere according to the sigma levels of 1.00, 0.99, 0.98, 0.96 0.93, 0.89, 0.85, 0.80, 0.75, 0.70, 0.65, 0.60, 0.55, 0.50, 0.45, 0.40, 0.35, 0.30, 0.25, 0.20, 0.15, 0.10, 0.05, and 0.00.

The topographic, vegetation, and land-water masks data were interpolated from the USGS global coverage with appropriate resolution for each domain: 10 min, 5 min, 30 sec for D1, D2, D3, and D4, respectively. The land use data were interpolated from the NCAR global coverage. The land use categories were obtained from FAO (Food and Agriculture Organization) and STATSGO (U.S. General Soil Map) with a spatial resolution of 30 sec. Soil moisture and temperature are initialized from the GFS global analysis with a resolution of 1°.

### 2.2. Episodes Analyzed

**Figure 2:**
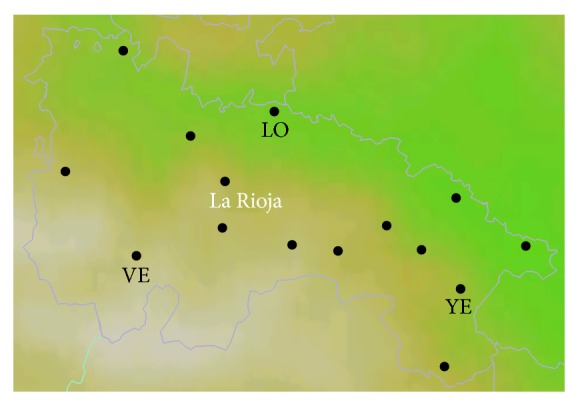
Location map of the weather stations used in the study.

Simulations were conducted in two periods of the year 2011, covering both the seasons of summer and winter. Both episodes, summer and winter, represent a synoptic situation with a weak pressure gradient: very high temperatures define the summer episode and, in contrast, in the winter episode there are relatively high temperatures while radiation levels are quite low. The analysis of the model was made in contrast to the data obtained from the network of meteorological stations owned by the Government of La Rioja, specifically, and as shown in Figure 2, the weather stations in Logroño (LO), Ventrosa (VE), and Yerga (YE). These stations record measurements of the following meteorological parameters.

- Air temperature.
- Global solar radiation.
- Rainfall.
- Relative humidity.
- Atmospheric pressure.
- Wind speed at 10 m.

### 2.3. Model Evaluation

In order to make a reliable study on the application of the weather-forecasting models to the field of wine production, the first step is to assess the models according to the desired application.

The large majority of weather model assessments focus on models of air quality studies and on how the models behave in the prediction of surface measurements of temperature, moisture, and precipitation. The typical assessment compares the specific measures with the results given by the model in a given space and time. Statistics such as the mean, the root mean square error, and the index of agreement were used to assess the performance of the model [41–46]. To directly compare and assess the observed data versus the model results, the following statistical values are presented in this study.

The mean balance error (see (1)) is defined as the average of the difference between the observed and predicted values and indicates the degree of over- or underprediction versus the observed values:

(1) $$\begin{array}{c} \text{MBE}=\frac{1}{N}\overset{N}{\underset{i=1}{\sum }}\emptyset_{i}. \end{array}$$

(i) The mean absolute error is
  (2) $$\begin{array}{c} \text{MAE}=\overset{N}{\underset{i=1}{\sum }}\frac{\left|\emptyset_{i}-\emptyset_{i\text{mon}}\right|}{N}, \end{array}$$
  where *∅*_*i*_ is the predicted value for a time *i*, *∅*_*i*mon_ is the monitored value for a time *i*, and *N* is the number of analyzed values.
(ii) Root mean square error (see (3)): both measures condense the difference between the observed and predicted values, but the RMSE is more sensitive to the extreme values than the MAE, due to the quadratic exponent:
  (3) $$\begin{array}{c} \text{RMSE}=\sqrt{\overset{N}{\underset{i=1}{\sum }}\frac{{\left(\emptyset_{i}-\emptyset_{i\text{mon}}\right)}^{2}}{N}}, \end{array}$$
  where *∅*_*i*_ is the predicted value for a time *i*, *∅*_*i*mon_ is the monitored value for a time *i*, and *N* is the number of analyzed values.
(iii) In addition, and although it is not very common that a measure of correlation between the observed and predicted values provides a clear indication of model behavior, its presence is normal in this kind of statistics. Therefore, the coefficient of determination (R2) is presented in this study, in order to show the variability between the data provided by the weather stations and the data given by the model.
(iv) Index of agreement-IOA [47, 48] is a measure that does provide information on the performance of the model under study and is defined as
  (4) $$\begin{array}{c} \text{IOA}=1-\frac{\sum_{i=1}^{N}{\left|\emptyset_{i}-\emptyset_{i\text{mon}}\right|}^{\alpha }}{\sum_{i=1}^{N}{\left(\left|\emptyset_{i}-\emptyset_{\text{mean}}\right|+\left|\emptyset_{i\text{mon}}-\emptyset_{\text{mean}}\right|\right)}^{\alpha }}, \end{array}$$
  where *∅*_*i*_ is the predicted value for a time *i*, *∅*_*i*mon_ is the observed value for a time *i*, *N* is the number of analyzed values, and *∅*_mean_ is the measure of the observations.

The adjustment index can be calculated for both *α* = 1 and *α* = 2. Both values are normalized and they indicate to what extent the deviations of the predicted values from the average of the observed values differ from the deviations of the observed values from the average thereof. Therefore, both values show the extent to which the model predictions are error-free, although the *d*_2_ is more sensitive because it is based on the quadratic differences versus the simple differences of the *d*_1_. Besides, this kind of statistical value allows making comparisons between different models, since the value is limited and relative.

### 2.4. Sensitivity Analysis

To perform the sensitivity analysis, the parameters adopted are the resolution of the domain and the mother domain, the interaction between domains, the resolution of the input data, and the boundary layer, as well as other parameters of the physical model. A summary of the different scenarios studied in the sensitivity analysis can be seen in Tables 1(a) and 1(b).

Each of these analyzed scenarios has been assessed on two different dates, one in summer and the other one in winter, in order to avoid a possible seasonality effect on the results.

#### 2.4.1. Sensitivity to the Domain Resolution

**Figure 3:**
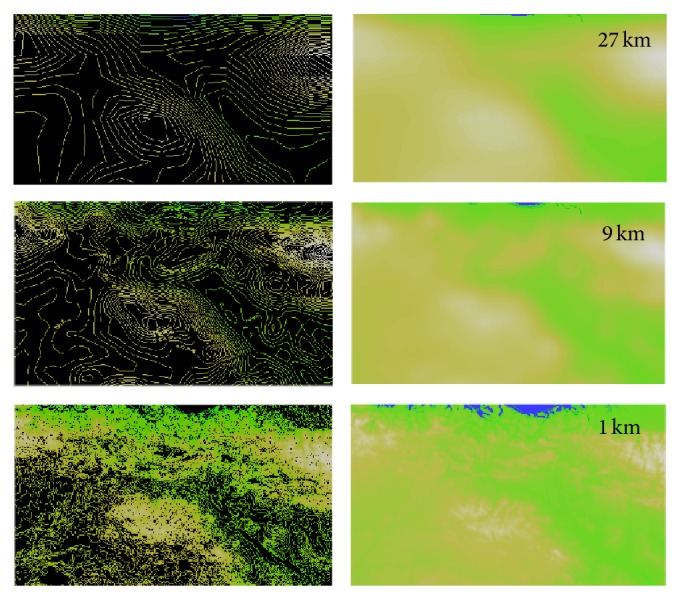
Example of different domains resolution.

One of the first parameters that must be analyzed when applying this type of model is the resolution of the domain used. In the specific case under study, the geographic location of the site (La Rioja, Spain) is characterized by a highly complex and diverse orography. Therefore, in order to obtain good results in the study of the soils of this region it is necessary to work with high resolutions in order to take into account all the constraints due to orography. The results are totally different when simulating winds, radiation, or temperature in La Rioja with a resolution of 27 km, 9 km, 3 km, or 1 km.  Figure 3 shows the graphic representation of the domain under study with different meshing resolutions, in particular 27, 9, and 1 km.

#### 2.4.2. Sensitivity to the Mother Domain

The MM5 is a mesoscale model which focuses on specific areas and has lateral limits defined by domains. The simulation results depend on the situation of the domains, and, therefore, a specific analysis has been made about this parameter over the course of this work. The first domain chosen is called the mother domain; the program will be limited to analyzing atmospheric phenomena that take place in that domain. In this way, two different mother domains have been chosen to study the sensitivity of the results.

**Figure 4:**
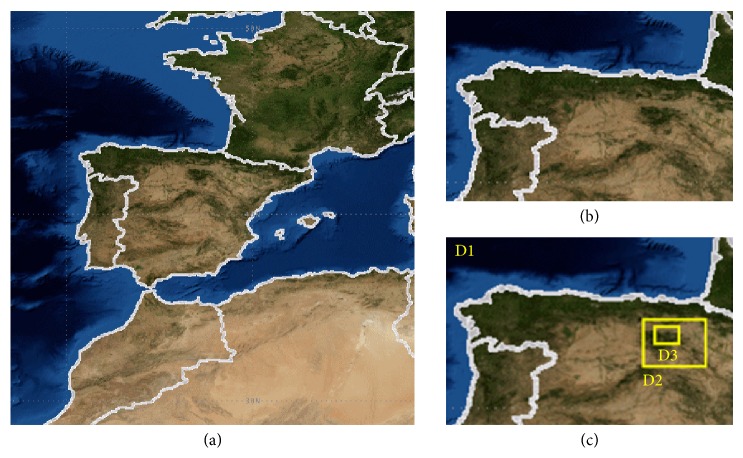
(a) Mother domain 1. (b) Mother domain 2. (c) Domains for* Logroño* with the mother domain 2.

- Mother domain 1: Spain along with parts of Europe and Africa (Figure 4(a)).
- Mother domain 2: northern Spain (Figure 4(b)).

Obviously, the larger the mother domain, the larger the scale phenomena that will be taken into account in the simulations, making it closer to the real situation. But, on the other hand, it is also necessary to ensure that the computational cost is as low as possible, and, therefore, a smaller domain is also selected to determine if the improvement in performance is significant at the level of application in the field of agriculture and viticulture.

The case of the mother domain 1 is the base-case model that has been used throughout the entire study and was described in Section 2.1 of methodology.

In the case of the mother domain 2, three mother domains have been defined. The first one, D1, corresponds to northern Spain. Within it, there is a domain D2 occupying the entire La Rioja region, with parts of neighboring communities. And as the last one, a domain D3 varies depending on the selected weather station (see Figure 4(c)).

- D1: 61 × 81 cells and 9 km resolution (mother domain 2).
- D2: 37 × 42 cells and 3 km resolution.
- D3: 34 × 49 cells and 1 km resolution.

#### 2.4.3. Sensitivity to the Interaction between Domains

Mesoscale models can work in a wide range of scales, from hundreds to tens of miles to just a few. Nesting techniques are used to take into account the characteristics of the study zones and they introduce into the model the synoptic information about the weather situation to be simulated. These techniques consist of the definition of an extensive low-resolution domain to simulate the most relevant synoptic constraints in a given situation. In the zone of interest, a nesting of the information of the external domain is made to solve the small-scale physical phenomena. In this new domain, the topography and the characteristics of the soil are described in greater detail, allowing the model to consider thermal and mechanical constraints that at lower resolutions are not considered. Thus, the results of the internal domain provide more accurate detailing of the atmospheric dynamics of the region under study.

There are two different techniques for this type of nesting. They differ depending on whether the information generated in the internal domain influences the external domain to improve the results. Thus, the one-way nesting or interaction between one-way domains transmits the information of the external domain to the internal one, and the latter solves the primitive equations. With this methodology, the simulations of the different domains are made in series, and the information is only transmitted from external domains to the internal ones.

On the other hand, the two-way nesting is characterized by the transfer of information between domains, influencing one another as physical equations are solved. Hence, the information of the internal domain influences the results of the external one. With this methodology, the domains are solved in parallel, exchanging information as equations are solved.

In general, two-way nesting is considered to be better, because it allows small scale phenomena to be transmitted to external domains and then influences the development of large-scale ones, coming as close as possible to reality. However, it can be said that the methodology contaminates in a certain way the results of the physical equations solved in external domains.

To carry out the analysis of the influence of the nesting technique in the MM5 results of its application in agriculture and viticulture, the rest of the model configuration has remained constant.

#### 2.4.4. Sensitivity to the Resolution of the Input Data

The current trend in meteorological models to increase the spatial resolution demands an improvement in the characterization territory to be studied. A detailed treatment of soil properties is increasingly important to capture the local mesoscale circulations induced by the thermal constraints of the soil [49]. The basic elements in the surface-atmosphere interaction are the energy and moisture exchange between the two systems. The moisture and heat flows from the earth's surface determine the distribution of the adjacent atmospheric layers of the air temperature, water vapor, precipitation, and cloud properties and, based on them, the radioactive flows from the atmosphere to the soil [50].

The MM5 allows analyzing the sensitivity of a mesoscale model to the properties of the soil to a maximum of 30 seconds (approximately 1 km). The program uses a simple scheme of characterization of the geophysical properties of the soil through a land use map widely used scheme in a great deal of mesoscale models [51] and uses the global map of United States Geological Survey (USGS) of land use. This map has been made from multitemporal data AVHRR-NDVI of 1 km of spatial resolution (1992-1993). The map works with 24 different categories of land use.

The influence of the resolution of the geophysical parameters of the soil obtained from the USGS map for a value of 2° (equivalent to 4 km) and 30′′ (equivalent to 1 km) is also analyzed. Aside from the USGS map of the geophysical properties of the soil, there are other sources with more categories than the ones displayed by the USGS. Among them, it is worth noting the CORINE, with 44 different categories, which allows for a greater degree of detail. The reason for having used the USGS map over the rest is its easy adaptation to the MM5 program; since it is not necessary to make any data transformation, and as noted in other studies, there have been no major improvements with the use of other maps.

#### 2.4.5. Sensitivity to the Boundary Layer (ABL)

Another Important aspect to consider is the sensitivity of the MM5 to the parameterization of the ABL. There are several studies analyzing the behavior of the MM5 model working with different parameterizations of the ABL. These studies focus mostly on analyzing the influence of the parameterization of the boundary layer on the results of meteorological character. The idea in the study is to analyze the impact of these variations in the results of their specific application to the field of new vineyard sites.

Within the MM5 model, there is the possibility of parameterization of the ABL in seven ways.

(0) None (none).
(1) Bulk PBL.
(2) High-resolution Blackadar.
(3) Burk-Thompson.
(4) Eta.
(5) MRF.
(6) Gayno-Seaman.
(7) Pleim-Chang.

For the present study, two different parameterizations of the ABL available in MM5 have been applied, because they lead to a lower computational cost and as it has been observed in previous studies, they are the ones with better results [52–54]. These parameterizations are

- ETA: Mellor-Yamada scheme used in the Eta model [55, 56]. It predicts the TKE (turbulent kinetic energy) and works with local vertical mixing. The computational cost is between the MRF scheme and the Blackadar,
- MRF: or Hong-PanABL, suitable for high resolution ABLs (as the Blackadar scheme). It is an efficient scheme based on the representation of Troen-Mahrt of the counter gradient end and the K profile in the mixture layer, as implemented in the MRF model of the NCEP (for further detail Hong and Pan's paper is recommended [57]).

The discussion of the results focuses on the domain D4, out of which conclusions can be drawn about the sensitivity of the MM5 and the convenience of working with any of the parameterizations. In order to analyze the sensitivity of the different models to the different parameterizations of the ABL, the remaining physical options of configuration have remained unchanged within the different simulations.

#### 2.4.6. Sensitivity to Other Parameters

There are multiple parameters to which the MM5 model is sensitive, apart from those analyzed in previous sections of this paper. Among them, for instance, it is worth noting the type of calculation of the radiation. Therefore, a short series of simulations have been made where the three different kinds of radiations have been tested.

- NONE: calculation of the radiation in cloudless conditions.
- CLOUD: calculation of the radiation considering cloudiness.
- RMTN: calculation of the radiation with complex cloudy conditions.

## 3. Results

**Figure 5:**
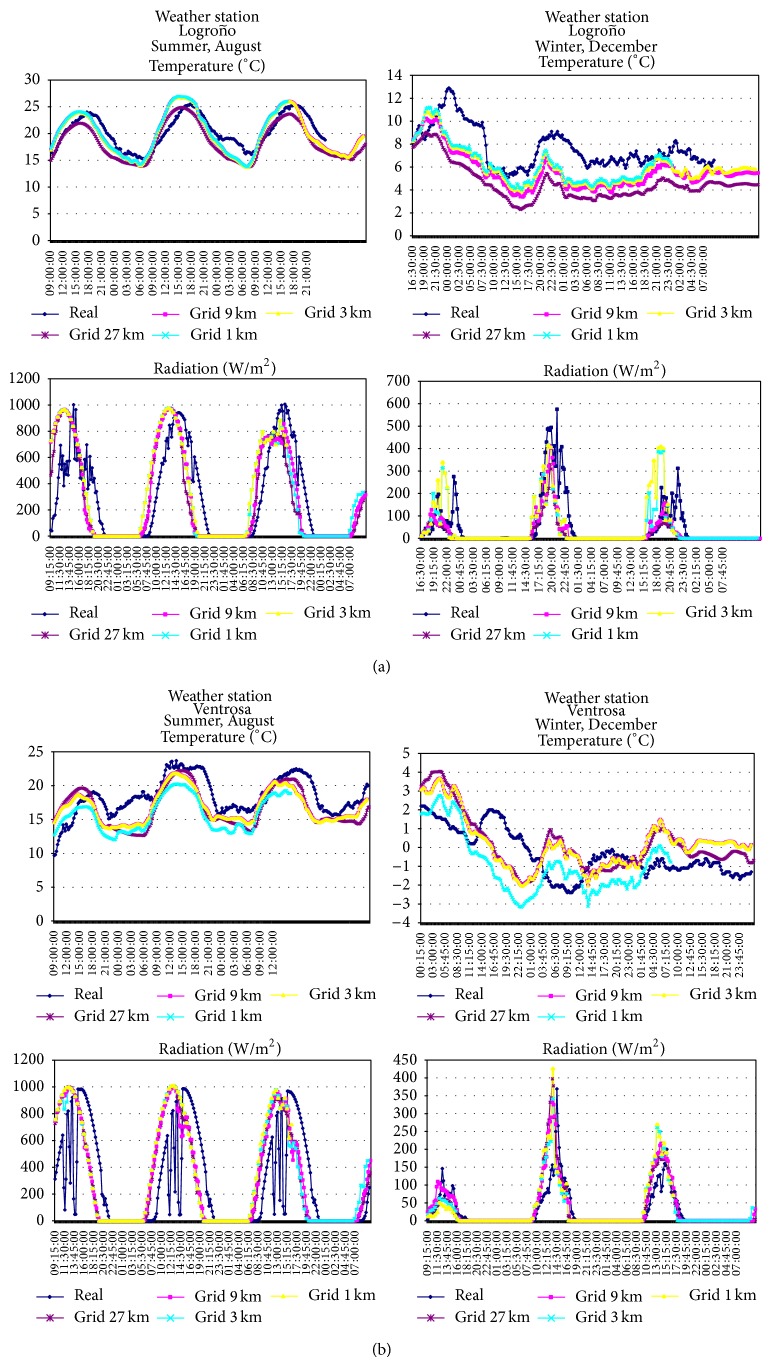
(a) Results obtained by the prediction model in the domains D1, D2, D3, and D4 and contrast with those obtained at the Logroño weather stations. (b) Results obtained by the prediction model in the domains D1, D2, D3, and D4 and contrast with those obtained at the Ventrosa weather stations.

Below, the results obtained by the generated model are presented, and, as indicated in Section 1.1, only the parameters of temperature and solar radiation have been simulated, which are then compared to the ones obtained in the weather station. The results shown in Figures 5(a) and 5(b) correspond to the model tested under a boundary layer ETA and four resolution domains, 27, 9, 3, and 1 km, respectively.

For each of the scenarios studied in each of the locations (Logroño, Ventrosa, and Yerga) the various statistics presented in Section 2 have been determined for each of the parameters analyzed: temperature at 2 m and solar radiation.

Table 2(a) presents the results obtained in the location Logroño for the base-case in each of the four domains that compose the model. Then, D1 is the initial mother domain with lower resolution (27 km) and the other three are the different nested domains (nested with respect to one another) and of higher resolution (9 km, 3 km, and 1 km, resp.). In addition, the results of the other alternative scenarios for the final domain of higher resolution in each case are presented.

From the results obtained, it can be seen that all the analyzed scenarios for the location of Logroño are, in average, colder during the winter (−0.33 K to −1.71 K), and all but one are on average warmer in the summer (0.27 K to 2.28 K). This bias to lower temperatures is, in part, the result to the reaction to the changes in air temperature on a synoptic scale of the deep soil temperature. For example, if cold temperatures remain over a period of several weeks, this will lead to a cooling of the deep soil temperature within the model. Then, when there is a change in the synoptic pattern to a period of warmer days, the deep soil temperature shows an excessively slow response at the time of becoming warmer. This increase of the temperature gradient between soil levels occurs mainly during daylight hours of heating and, therefore, the corresponding heat flow from the upper to the lower layers of the soil system of the model is increased too. Due to this increase, the heat flow that the model simulates between the soil surface layer and the lower layers of the atmosphere is reduced, and as a result, the predicted temperature at 2 m over the ground surface is lower than expected. The only exception is the one seen when using the ABL type ETA, which has an average performance that is cooler in summer (−0.10 K).

**Figure 6:**
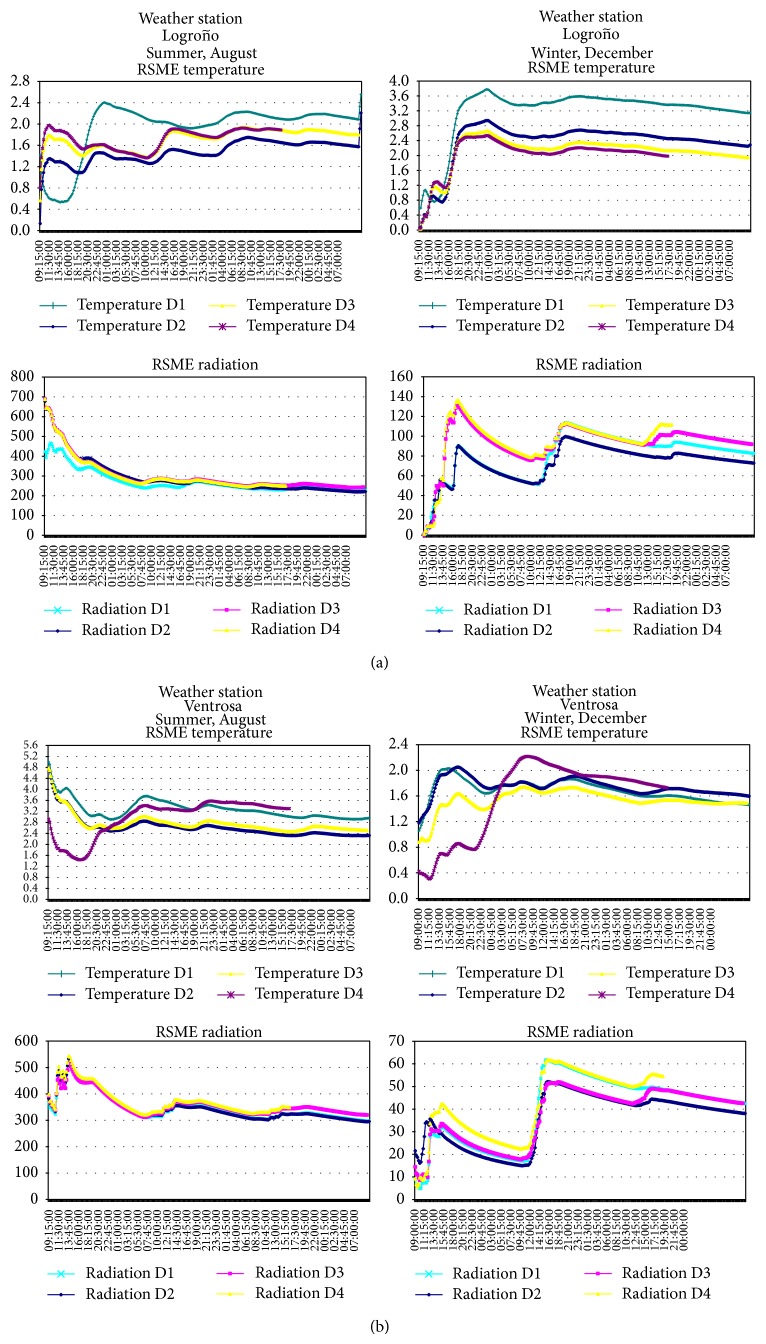
(a) Root mean square error (RMSE) obtained by the application of the model in* Logroño*. (b) Root means square error (RMSE) obtained from the application of the model in Ventrosa.

The root mean square error (RMSE) of the temperature decreases in winter with respect to summer in most of the simulations (Table 2(a) and Figure 6(a)). The simulated temperature at 1 km has a RMSE higher than the corresponding RMSE to 3 km and 9 km in summer, slightly improving in winter. It may suggest that there is not a significant improvement in terms of soil temperature in the surface despite the improvement in the meshing model. Gego et al. [41] analyzed the improvement in temperature simulations at 2 m above the ground, reducing the size of the meshing from 12 to 4 km, which leads to overall improvement at noon and early afternoon hours, but with a slight worsening in the first and last hours of the morning. Mass et al. [44] also found slight improvements when reducing the meshing from 36 to 12 km, but very little improvement when reducing from 12 to 4 km.

Besides, to put the obtained results into perspective, it is good to analyze other studies, such as the one presented by Baker [58] who found a daily variability for the MAE of between 1.50 K and 3.00 K in the data of a full year. Mass et al. [44] showed similar statistics for a prediction made with MM5 over the northwest of the United States, with a particularly complex topography; its MAE remained near 2.25 K.

These errors in the prediction of the temperature at 2 m are not significant in any way as they do not affect in a visible way the evolution of the vine in the growing period or in the summer maturation periods, since the temperature margin is within the established range, as exposed in Section 1.1.

Finally, in the case of the simulation of the solar radiation, the range of the MAE goes from 50 to 65 w/m^2^. An improvement is also seen to increase in the resolution, both in the MAE and in the IOA. In the remaining alternative scenarios, a more marked effect can be seen when altering the ABL with higher error rates in the summer and better results in winter. Given the limits of sun exposure required for growing vine presented in Section 1.1, the model is completely valid in both cases, as the margins of solar radiation required by the vine are quite large and the error made by the model is insignificant compared to those values required for the maturation of the grapes.

Furthermore, as presented in Table 2(a), there are no shown results for the alternative scenario that analyzes the modification of other parameters such as the type of calculation employed for the radiation. This happens because, in all the locations, the results obtained in the case of temperature have been very similar. In contrast, in the radiation, as expected, there is a slight improvement from the type CLOUD to NONE, since taking into account the clouds leads to more reliable data. For type RRTM there has not been any improvement observed, and given the resource consumption, it should be considered whether or not to study its use depending on the specific application desired.

In Table 2(b) and the RMSE graphs of Figure 6(b), the results obtained for the location of Ventrosa are shown, the same being completely similar to those obtained in Logroño. This demonstrates the validity of the model in uneven geographic locations, such as the case with a valley of a river basin (Logroño) and a mountainous plateau (Ventrosa).

The results obtained for the location of Yerga are not shown because they are completely similar to those obtained in Logroño and Ventrosa.

## 4. Discussion

A mesoscale model has been presented for the location of ideal sites for new vineyard plantations within the Rioja Qualified Denomination of Origin (Spain), in order to improve the final quality of the wine, as the vine alone cannot guarantee a good grape production, much less quality wine. It is necessary to take into account the interaction of other factors that influence the proper maturation. Among them, the so-called variable factors that are difficult to control but are predictable, and, therefore, it is possible to determine the locations with optimal conditions of temperature, radiation, and rainfall for new plantations.

Throughout this paper, the performance of the mesoscale model MM5 has been analyzed for different kinds of parameterizations, with resolutions of 27, 9, 3, and 1 km, respectively, applied to the prediction of ideal environmental conditions for new vine plantations in the Rioja Qualified Denomination of Origin. The concrete development has been focused on analyzing the region of La Rioja (Spain), characterized for having both zones with simple orography (flat areas) and zones with complex orography (mountainous areas). The quality of the outputs is a critical factor for the success of the model; therefore, the work has been made with a data resolution as accurate as possible, and, in as much detail, adjusting to the maximum of the solar and thermal conditions of specific areas under study. Thus, the model simulations have been made with the highest possible degree of resolution possible, which, in the case of MM5, is 1 km.

Specifically, two episodes have been analyzed: one in summer season and the other in the winter, and the results have been compared with three weather stations located in different areas of La Rioja. The results obtained in the modeling largely resemble those actually observed in the weather stations of Logroño, Ventrosa, and Yerga.

To test the validity of the results, these have been characterized by statistics such as the MBE, ABSE, RMSE, R2, and IOA. It can be seen that the RMSE of the temperature decreases in winter with respect to the summer in most of the simulations. The temperature simulated at 1 km has an RMSE higher than the corresponding RMSE to 3 km and 9 km in summer, slightly improving in winter. These errors in the prediction of the temperature at 2 m are insignificant since due to its range of values they do not appreciably affect the evolution of the vine in the growing period or in the summer period of maturation.

For the simulation of the solar radiation, the MAE ranges from 50 to 65 w/m^2^, showing an improvement with the increase in resolution both in the MAE and in the IOA. In the remaining alternative scenarios, it can be observed as a more marked effect when the ABL is altered, with higher error rates in the summer and better results in winter, taking into account the minimum annual sun exposure hours required.

As a final conclusion, it can be asserted that the model is completely valid for the analyzed cases, and, therefore, it can be applied to the entire viticulture region, constituting then a remarkable analysis tool to select the ideal location of new vineyard plantations.

## Figures and Tables

**(a):** 

	Domains			Physical parameters					Boundary conditions			Resolution
Studies		Resolution and size		Cumule	PBL	Humidity diagram	Radiation diagram	Soil diagram	Lateral limit	Upper limit	Nesting technique	Input data
		Horizontal	Vertical		BLTYP	IMPHYS	FRAD	ISOIL	IUDY	IFPUR		NTYPE
Base- case resolution domain	D1	86 × 20 27 Km	23	Grell 3	MRF 5	Simple ice 4	Cloud 2	Five-layer 1	Relaxation/inflow-outflow 3	Upper radiative condition 1	1-way	19 Km 3
	D2	100 × 100 9 Km	23	Grell 3	MRF 5	Simple ice 4	Cloud 2	Five-layer 1	Time-dependent/Nest 2	Upper radiative condition 1	1-way	9 Km 4
	D3	43 × 55 3 Km	23	None 1	MRF 5	Simple ice 4	Cloud 2	Five-layer 1	Time-dependent/Nest 2	Upper radiative condition 1	1-way	0,9 km 6
	D4	31 × 40 1 Km	23	None 1	MRF 5	Simple ice 4	Cloud 2	Five-layer 1	Time-dependent/Nest 2	Upper radiative condition 1	1-way	0,9 km 6

Boundary layer	D1	86 × 20 27 Km	23	Grell 3	**ETA/MRF ** **4/5**	Simple ice 4	Cloud 2	Five-layer 1	Relaxation/inflow-outflow 3	Upper radiative condition 1	1-way	19 Km 3
	D2	100 × 100 9 Km	23	Grell 3	**ETA/MRF ** **4/5**	Simple ice 4	Cloud 2	Five-layer 1	Time-dependent/Nest 2	Upper radiative condition 1	1-way	9 Km 4
	D3	43 × 55 3 Km	23	None 1	**ETA/MRF ** **4/5**	Simple ice 4	Cloud 2	Five-layer 1	Time-dependent/Nest 2	Upper radiative condition 1	1-way	0,9 Km 6
	D4	31 × 40/1 Km	23	None 1	**ETA/MRF ** **4/5**	Simple ice 4	Cloud 2	Five-layer 1	Time-dependent/Nest 2	Upper radiative condition 1	1-way	0,9 Km 6

Radiation diagram	D1	86 × 20 27 Km	23	Grell 3	MRF 5	Simple ice 4	**None-cloud RRTM **	Five-layer 1	Relaxation/inflow-outflow 3	Upper radiative condition 1	1-way	19 Km 3
	D2	100 × 100 9 Km	23	Grell 3	MRF 5	Simple ice 4	**None-cloud RRTM **	Five-layer 1	Time-dependent/Nest 2	Upper radiative condition 1	1-way 2-way	9 Km 4
	D3	43 × 55 3 Km	23	None 1	MRF 5	Simple ice 4	**None-cloud RRTM **	Five-layer 1	Time-dependent/Nest 2	Upper radiative condition 1	1-way 2-way	0,9 Km 6
	D4	31/40 1 Km	23	None 1	MRF 5	Simple ice 4	**None-cloud RRTM **	Five-layer 1	Time-dependent/Nest 2	Upper radiative condition 1	1-way 2-way	0,9 Km 6

**(b):** 

	Domains			Physical parameters					Boundary conditions			Resolution
Studies		Resolution and size		Cumule	PBL	Humidity diagram	Radiation diagram	Soil diagram	Lateral limit	Upper limit	Nesting technique	Input data
		Horizontal	Vertical		BLTYP	IMPHYS	FRAD	ISOIL	IUDY	IFPUR		NTYPE
Input Resolution	D1	86 × 20 27 Km	23	Grell 3	MRF 5	Simple ice 4	Cloud 2	Five-layer 1	Relaxation/inflow-outflow 3	Upper radiative condition 1	1-way	**19 Km** ** 3**
	D2	100 × 100 9 Km	23	Grell 3	MRF 5	Simple ice 4	Cloud 2	Five-layer 1	Time-dependent/Nest 2	Upper radiative condition 1	1-way	**9 Km** **4**
	D3	43 × 55 3 Km	23	None 1	MRF 5	Simple ice 4	Cloud 2	Five-layer 1	Time-dependent/Nest 2	Upper radiative condition 1	1-way	**4 Km** **6**
	D4	31 × 40 1 Km	23	None 1	MRF 5	Simple ice 4	Cloud 2	Five-layer 1	Time-dependent/Nest 2	Upper radiative condition 1	1-way	**4 Km** **6**

Mother domain	D1	**61 × 81 ** **9 Km**	23	Grell 3	MRF 5	Simple ice 4	Cloud 2	Five-layer 1	Relaxation/inflow-outflow 3	Upper radiative condition 1	1-way	19* *Km 3
	D2	**37 × 42 ** **3 Km**	23	Grell 3	MRF 5	Simple ice 4	Cloud 2	Five-layer 1	Time-dependent/Nest 2	Upper radiative condition 1	1-way	9* *Km 4
	D3	**34 × 49 ** **1 Km**	23	None 1	MRF 5	Simple ice 4	Cloud 2	Five-layer 1	Time-dependent/Nest 2	Upper radiative condition 1	1-way	0,9* *Km 6

Nesting technique	D1	86 × 20 27 Km	23	Grell 3	MRF 5	Simple ice 4	Cloud 2	Five-layer 1	Relaxation/inflow-outflow 3	Upper radiative condition 1	**1-way**	19* *Km 3
	D2	100 × 100 9 Km	23	Grell 3	MRF 5	Simple ice 4	Cloud 2	Five-layer 1	Time-dependent/Nest 2	Upper radiative condition 1	**1-way ** **2-way**	9* *Km 4
	D3	43 × 55 3 Km	23	None 1	MRF 5	Simple ice 4	Cloud 2	Five-layer 1	Time-dependent/Nest 2	Upper radiative condition 1	**1-way ** **2-way**	0,9* *Km 6
	D4	31/40 1 Km	23	None 1	MRF 5	Simple ice 4	Cloud 2	Five-layer 1	Time-dependent/Nest 2	Upper radiative condition 1	**1-way ** **2-way**	0,9* *Km 6

**(a):** 

	Summer					Winter				
	MBE	MAE	RMSE	R2	IOA	MBE	MAE	RMSE	R2	IOA
Temperature										
BC_D1	0.27	1.28	1.56	0.95	0.96	−1.71	1.73	1.95	0.76	0.76
BC_D2	1.42	1.87	2.43	0.95	0.91	−0.74	1.06	1.28	0.69	0.87
BC_D3	1.85	2.10	2.71	0.96	0.89	−0.45	0.99	1.18	0.68	0.89
BC_D4	2.00	2.18	2.81	0.96	0.88	−0.33	0.95	1.13	0.69	0.90
Mother domain 2 (D3)	2.28	2.47	2.98	0.96	0.87	−0.57	1.26	1.40	0.66	0.88
Domain inter (D4)	2.04	2.20	2.84	0.96	0.88	−0.33	0.95	1.14	0.69	0.90
Input data (D4)	2.05	2.21	2.84	0.96	0.88	−0.33	0.96	1.14	0.68	0.90
ABL (D4)	−0.10	1.14	1.44	0.91	0.96	−1.44	1.56	1.79	0.70	0.79
Radiation										
BC_D1	34.05	63.95	123.06	0.91	0.97	−40.72	43.34	95.19	0.60	0.70
BC_D2	36.19	64.86	123.95	0.91	0.97	−26.10	49.40	99.80	0.35	0.68
BC_D3	42.00	56.37	117.21	0.92	0.97	−9.79	56.07	107.22	0.27	0.69
BC_D4	42.00	56.16	116.94	0.92	0.97	−2.76	54.55	106.00	0.31	0.72
Mother domain 2 (D3)	40.33	58.03	118.75	0.92	0.97	−2.43	53.04	107.31	0.29	0.71
Domain inter (D4)	42.00	56.12	116.95	0.92	0.97	−3.41	55.13	107.31	0.30	0.71
Input data (D4)	41.90	56.11	116.92	0.92	0.97	−3.89	54.76	106.53	0.30	0.71
ABL (D4)	33.31	65.06	125.56	0.90	0.97	−1.74	49.58	100.64	0.40	0.78

**(b):** 

	Summer					Winter				
	MBE	ABSE	RMSE	R2	IOA	MBE	ABSE	RMSE	R2	IOA
Temperature										
BC_D1	−2.42	3.47	4.44	0.47	0.65	0.58	0.72	0.99	0.79	0.86
BC_D2	−0.89	2.44	3.06	0.50	0.77	2.25	2.25	2.46	0.67	0.55
BC_D3	−1.25	2.55	3.30	0.50	0.75	2.10	2.11	2.38	0.61	0.56
BC_D4	−1.28	2.55	3.32	0.51	0.75	2.23	2.27	2.57	0.55	0.53
Mother domain 2 (D3)	−0.45	2.42	3.01	0.55	0.79	2.39	2.45	2.71	0.55	0.52
Domain inter (D4)	−2.48	2.76	3.68	0.54	0.70	0.44	0.87	1.05	0.61	0.83
Input data (D4)	−2.65	2.91	3.90	0.53	0.68	0.47	0.85	1.03	0.62	0.83
ABL (D4)	−3.10	3.12	3.39	0.74	0.68	−0.32	0.73	0.84	0.82	0.90
Radiation										
BC_D1	62.60	95.71	230.13	0.70	0.91	11.61	27.57	53.14	0.63	0.85
BC_D2	55.35	98.37	230.41	0.69	0.91	23.41	32.69	62.89	0.64	0.82
BC_D3	64.41	94.64	235.46	0.69	0.91	25.56	33.66	64.67	0.65	0.81
BC_D4	65.14	93.23	234.07	0.69	0.91	53.53	57.94	110.20	0.69	0.69
Mother domain 2 (D3)	58.16	99.13	236.91	0.68	0.90	27.08	33.24	62.81	0.71	0.83
Domain inter (D4)	71.20	91.17	235.18	0.70	0.91	33.90	43.76	89.93	0.65	0.74
Input data (D4)	71.46	91.15	235.22	0.70	0.91	33.61	43.74	89.79	0.64	0.74
ABL (D4)	69.25	95.87	239.08	0.68	0.90	6.66	23.65	48.08	0.63	0.86
